# Effects of wogonin on the growth and metastasis of colon cancer through the Hippo signaling pathway

**DOI:** 10.1080/21655979.2021.2019173

**Published:** 2022-01-17

**Authors:** Wenli You, Aiting Di, Lize Zhang, Gang Zhao

**Affiliations:** Department of Chinese Medicine, The Affiliated Hospital of Qingdao University, Qingdao, China

**Keywords:** Wogonin, colon cancer, Hippo signaling, epithelial-mesenchymal transition, IRF3

## Abstract

Wogonin is an effective component of *Scutellaria baicalensis* Georgi, which exhibits anti-tumor activity. The aim of this study was to explore the effects of wogonin on colon cancer (CC). Human CC cell lines, SW480 and HCT116, were cultured, and MTT assay was performed to detect cell survival. RT-qPCR and Western blotting were used to measure mRNA and protein expression, respectively. The migration and invasion abilities of the CC cells were determined by a transwell assay. Immunofluorescence staining was performed to determine the localization of IRF3. Xenograft mice were used to investigate the effects of wogonin on CC *in vivo*. Wogonin inhibited the survival and metastasis of CC cells. In addition, wogonin suppressed epithelial-mesenchymal transition (EMT). Furthermore, the protein expression of YAP1 and IRF3 was downregulated, and p-YAP1 was upregulated after wogonin treatment. Wogonin also suppressed IRF3 expression in the nuclei of CC cells and overexpression of YAP1 reversed the effects of wogonin in CC cells. Finally, wogonin inhibited the tumor growth in the mice and overexpression of YAP1 reversed the wogonin effects. Thus, these results showed that wogonin relieved the carcinogenic behaviors and EMT of CC cells via the IRF3-mediated Hippo signaling pathway.

## Introduction

Colon cancer (CC) is the fifth deadliest cancer worldwide [1]. It is estimated that CC accounts for approximately seven hundred thousand deaths annually, 5.8% of all cancer deaths [2]. Multiple factors are closely related to the progression of colon cancer, including the activation of oncogenes and inactivation of tumor suppressor genes [3]. In developing countries, the incidence rate is steadily increasing [4]. Approximately 60% of patients with CC are prone to distant metastasis, which leads to high mortality [5]. In spite of advances in screening and treatments, such as surgery and surgery combined with chemotherapy and radiotherapy, the median survival rate of CC is still very poor [6]. Consequently, to enhance the effectiveness of treatments for patients with CC, it is imperative to identify an effective drug for suppressing the occurrence and development of CC.

*Scutellaria baicalensis* Georgi (SBG) is a traditional Chinese herbal medicine that has been widely applied in the treatment of inflammation [7], and bacterial [8] and viral infections [9] in China and Western countries. In addition, SBG has been confirmed to play an anti-tumor role in various tumors *in vivo* and *in vitro* [10,11]. Wogonin (C_15_H_10_O_5_) is an effective component of SBG and exhibits anti-viral and neuroprotective activities [12]. Furthermore, many studies have shown that wogonin inhibits the growth, metastasis, and infiltration ability of cancer cells to alleviate the occurrence and development of various tumors, such as in ovarian cancer [13], hepatoma cells [14], and breast cancer cells [15]. However, reports on the effects of wogonin in CC are limited.

Previous reports have confirmed that the anti-tumor mechanism of wogonin involves multiple signaling pathways [13,15]. In cancer cells, the Hippo signaling pathway regulates the balance of cell proliferation and apoptosis by participating in the control of cell division, thus regulating organ volume [16]. Yes-associated protein (YAP), an important molecule in the Hippo signaling pathway, is widely activated in human malignant tumors [16]. Many external and internal factors related to cancer jointly activate the microenvironment that inhibits YAP in normal tissues, including mechanical transduction, inflammation, oncogenic signals, and changes in upstream signaling molecules [17,18]. Non-phosphorylated YAP plays an important role in the nucleus; as a co-transcription factor, YAP binds to related transcription factors and initiates the transcription of downstream related target genes to promote cell proliferation and organ growth. On the other hand, phosphorylated YAP is transferred out of the nucleus and blocked in the cytoplasm, thus inhibiting the transcription of YAP-related proliferation target genes [19].

As a key transcription factor of the innate immune response, interferon regulatory factor 3 (IRF3) plays an important role in the resistance and control of viral infections [20]. IRF3 is located in the cytoplasm in the form of an inactive monomer and its expression is stable and persistent in most cells. When the DNA is damaged, IRF3 is phosphorylated by DNA-dependent protein kinase, which is involved in apoptosis induced by DNA damage, and migrates from the cytoplasm to the nucleus [21]. A recent study confirmed that IRF3 participates in the regulation of YAP1 expression. As an agonist of YAP, IRF3 was confirmed to be a therapeutic target in gastric cancer [22]. However, the role of IRF3 in CC remains unclear.

Therefore, in the present study, we aimed to explore the effects of wogonin on the growth and metastasis of CC cells. We hypothesized that wogonin may relieve the carcinogenic behaviors of CC cells via the IRF3-mediated Hippo signaling pathway.

## Materials and methods

### Cell culture

The human CC cell lines SW480 and HCT116 were provided by the Cell Bank of the Chinese Academy of Sciences (Shanghai, China). The cells were cultured in Dulbecco’s modified Eagle’s medium (DMEM) containing 10% fetal bovine serum at 37°C and under 5% CO_2_, and passaged every 2–3 days. The cells were randomly divided into control, 0.5 μM wogonin, 1 μM wogonin, 2 μM wogonin group, and 4 μM wogonin groups, and cultured for 24 h for the following experiments. The study protocol was approved by the Ethics Committee of the Affiliated Hospital of Qingdao University (No. 2020–86943). Written informed consent was obtained from all participants prior to the study.

### MTT assay

As described by a previous study [23], The cells were seeded in a 96-well culture plate. Four hours before the end of the culture, 10 μL of MTT was added. After culturing, the medium was aspirated and 150 μL DMSO was added to each well. The absorbance (A) value was measured using an enzyme-linked immunoassay at 490 nm. The cell survival rate of the control group was 100%, and the cell survival rate of the remaining groups was calculated as follows: (A value of the experimental group/A value of the control group) ×100%.

### Transwell assays

According to a previous study [24], the cells were seeded on a Transwell plate; 100 μL of cell suspension containing 1% serum (1 × 10^4^ cells/well) was added to the upper chamber, and 15% FBS and 10 mg/L fibronectin were added to the lower chamber (with Matrigel-coated membrane for migration, not coated with Matrigel for invasion). The cells were then placed in a CO_2_ incubator (37°C, 5% CO_2_) for 48 h, after which the cells were removed and stained with 0.5% crystal violet for 15 min. The average number of cells in five randomly selected fields of view observed under the microscope represented the number of invading or migrating cells.

### Western blot

According to a previous study [25], the proteins of the cells were extracted and quantified using the BCA protein concentration assay kit. SDS-PAGE vertical plate electrophoresis was performed, and electroporation was performed on a PVDF membrane. The membrane was then sealed with skim milk powder for 2 h and washed with PBST for 10 min. Next, the PVDF membrane was incubated with the primary antibody (YAP1, 1:1000; p-YAP1, 1:500; IRF3, 1:800; β-tubulin, 1:1000) at 4°C overnight. Then, the PVDF membrane was incubated with horseradish peroxidase-labeled secondary antibody (1:500) and incubated at room temperature for 2 h. ECL reagent and Bio-Rad system were used for measuring the luminescence. Bio-Rad-Image-Lab software was used to analyze the gray value of the bands. β-tubulin was used as the internal reference. The relative protein expression level was expressed as the ratio of the gray value of each target band to the gray value of the internal reference.

### Immunofluorescence

According to a previous study [26], the cells were fixed with 4% paraformaldehyde and 0.3% Triton X-100 was used to perforate the cell membranes. Then, the cells were blocked and stained with anti-IRF3 antibody (GeneTex, USA) at 4°C overnight. Next, the cells were washed and cultured with FITC-conjugated secondary antibodies (Bioworld, USA) for 2 h, following which, the cells were stained with DAPI for 4 min. Finally, an FV3000 laser scanning confocal microscope was used to capture the images.

### Cell transfection

According to a previous study [27], siRNAs targeting IRF3 (si-IRF3 1#, si-IRF3 2#, and si-IRF3 3#), siRNA targeting negative control (si-nc), overexpression of YAP1 (oe-YAP1), and oe-nc were purchased from GenePharma (Shanghai, China). Lipofectamine 3000 (Invitrogen, USA) was used to transfect the CC cells. After 48 h of transfection, cells were selected for further analysis.

### Xenograft tumor formation

Nude BALB/c mice (20 ± 2 g) were purchased from the Animal Center of Nanjing Medical University. According to a previous study [28], SW480 cells were cultured in the medium for 24 h until 80% confluency was achieved. The cells in the logarithmic growth phase were then digested with pancreatin, inoculated into 6-well culture plates, and incubated with oe-nc and oe-YAP1 for transfection. SW480 cells (2 × 10^8^ cells/mL) were injected subcutaneously into the mice. On the 8th day after inoculation, the mice were randomly divided into control (intraperitoneal injection of normal saline), wogonin group, wogonin+oe-nc, and wogonin+oe-YAP1 (intraperitoneal injection of 2 μM wogonin) groups, with six mice in each group. Wogonin and normal saline were administered once every other day, for 10 days.

A Vernier caliper was used to measure the long (L) and short (S) diameters of the tumor once a week, and the volume (V) was calculated using the following formula: V = 0.5 × L× W^2^. The volume was measured weekly. Four weeks later, the mice were anesthetized by intraperitoneal injection of 3% pentobarbital (160 mg/kg) and sacrificed. The tumor weight was measured.

### RT-qPCR

TRIzol (Shanghai Pufei Biotech Co., Ltd) was used to extract and purify the total RNA from the tissues according to the manufacturer’s instructions, and cDNA was synthesized using the Reverse Transcription Kit (TAKARA). Gene expression was detected using the SYBR Master Mix Kit (TAKARA) in Real-Time PCR platform LightCycler480 (Roche), and the reaction conditions were: 95°C for 30s; 40 cycles of 95°C for 5s and 60°C for 30s; and dissociation at 95°C for 15s, 60°C for 30 s, and 95°C for 15s. The relative gene expression levels were calculated using the 2^−ΔΔCt^ method [29]. GAPDH was used as the internal reference.

## Statistical analysis

The data in the current study are presented as the mean ± standard deviation (SD), and the statistical significance of the differences between groups was examined using the Student’s t-test, and analysis of variance (ANOVA) was used for the comparison among multiple groups. All data were processed using Microsoft Office Excel 2016, and charts were drawn using GraphPad Prism (Version 8.0.2). Statistical significance was set at P < 0.05.

## Results

This study confirmed that wogonin inhibited expressions of YAP1, and the YAP1 targeted genes including AXL, CYR61 and CTGF in CC cells. Additionally, wogonin suppressed EMT development and the carcinogenic process of CC through the IRF3-mediated Hippo signaling pathway. Our results provide novel insights into the promotion and application of wogonin, which is expected to be a novel therapy for the treatment of CC.

### Wogonin decreased the cell survival rates of CC cells

**Figure 1.**
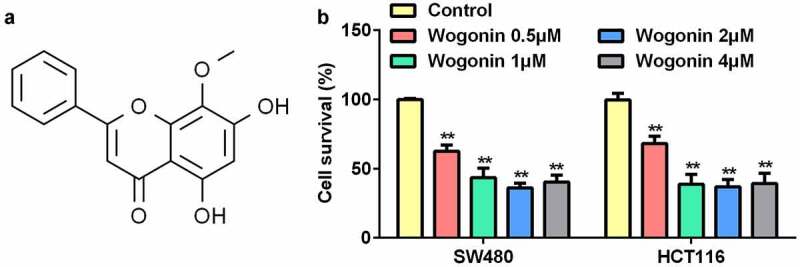
Effect of wogonin on the survival of CC cells.

First, we explored the effects of wogonin on the survival rates of CC cells using the MTT assay. (Figure 1a) shows the molecular structure of wogonin. The results showed that wogonin significantly suppressed the cell survival rates of CC cells in a dose-dependent manner; the survival rate in the 4 μM wogonin group was higher than that in the 2 μM wogonin group (Figure 1b). Thus, we selected 0.5, 1, and 2 μM wogonin for subsequent experiments.

(a) Molecular structure of wogonin. (b) The MTT assay was performed to detect cell survival. **P < 0.01, vs. Control group.

### Wogonin inhibited the migrated and invaded cells and regulated the protein expression of epithelial-mesenchymal transition (EMT)-related genes in CC cells

**Figure 2.**
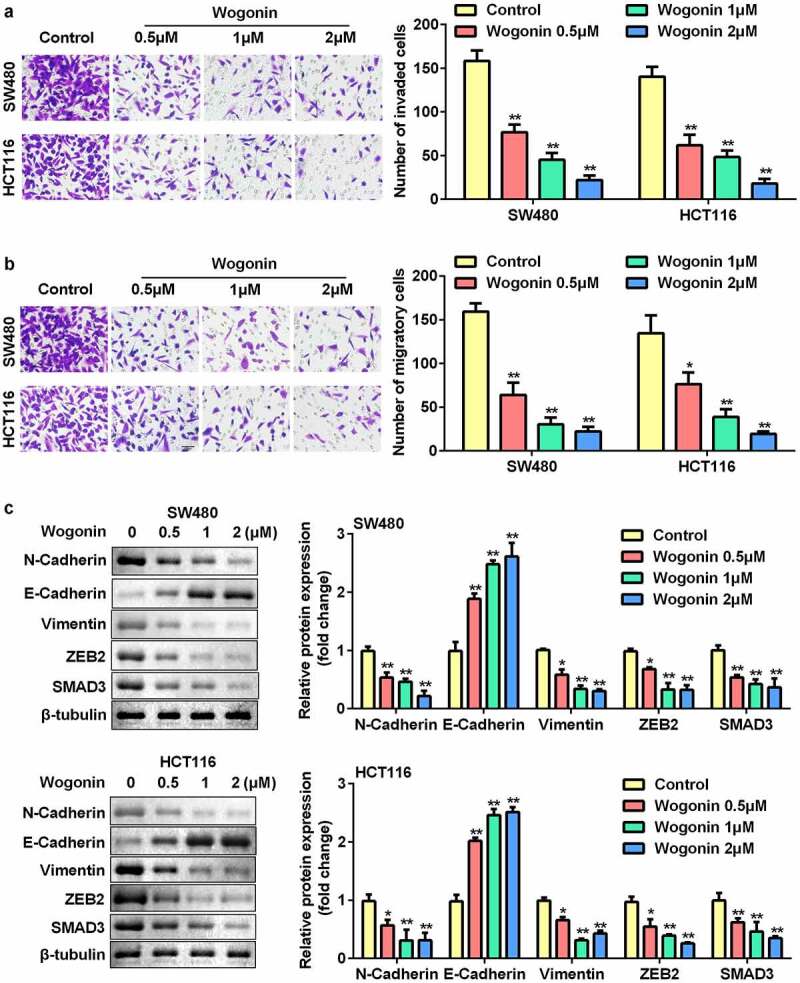
Effects of wogonin on metastasis and protein expression of EMT-related genes of CC cells.

We further assessed the effects of wogonin on migration, invasion, and protein expression of EMT-related genes in CC cells. The results showed that wogonin significantly decreased the number of migrating and invading cells in a dose-dependent manner (Figure 2a–b). In addition, wogonin significantly upregulated the expression of E-cadherin and downregulated the expression of vimentin, ZEB2, N-cadherin, and SMAD3 (Figure 2c).

(a–b) The invasion and migration ability of CC cells was measured using the Transwell assay. (c) The protein expression of E-cadherin, Vimentin, ZEB2, N-cadherin, and SMAD3 were determined by Western blotting. *P < 0.05, **P < 0.01, vs. Control group.

### Wogonin decreased YAP1 protein expression in CC cells

**Figure 3.**
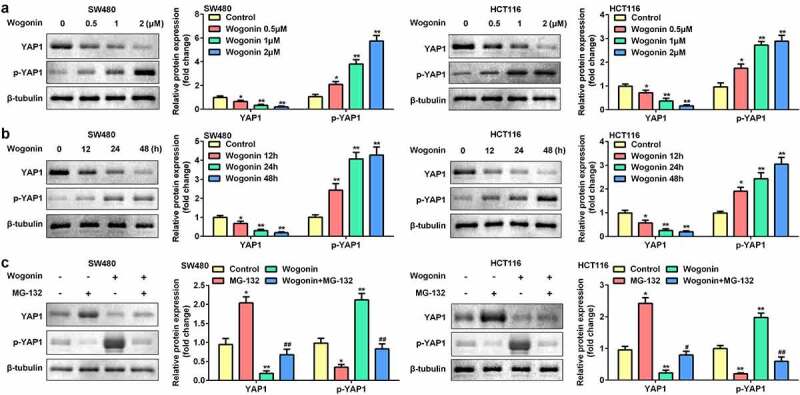
Effect of wogonin on the protein expression of YAP1 and p-YAP1 in CC cells.

Next, the effects of wogonin on the protein expression of YAP1 and p-YAP1 were measured. The protein expression of YAP1 was significantly downregulated, and that of p-YAP1 was upregulated after wogonin treatment in a dose-dependent manner (Figure 3a). Consequently, we selected 2 μM wogonin for subsequent experiments. The results showed that wogonin significantly decreased YAP1 expression and increased p-YAP1 expression in a time-dependent manner (Figure 3b). Next, the CC cells were treated with the proteasome inhibitor MG-132; MG-132 significantly increased YAP1 expression and decreased p-YAP1 expression in CC cells. However, MG-132 reversed the effects of wogonin on the protein expression of YAP1 and p-YAP1 (Figure 3c). Therefore, 2 μM wogonin and 24 h processing time were selected for subsequent experiments.

(a) CC cells were treated with 0.5, 1, and 2 μM wogonin, and the protein expression of YAP1 and p-YAP1 was detected by Western blotting. (b) CC cells were treated with 2 μM wogonin for 12, 24, and 48 h, and the protein expression of YAP1 and p-YAP1 was detected by Western blotting. (c) CC cells were treated with wogonin and MG-132, and the protein expression of YAP1 and p-YAP1 was detected by Western blotting. *P < 0.05, **P < 0.01, vs. Control group. #P < 0.05, ##P < 0.01, vs. Wogonin group.

### Wogonin decreased the protein expressions of YAP1 targeted genes in CC cells

**Figure 4.**
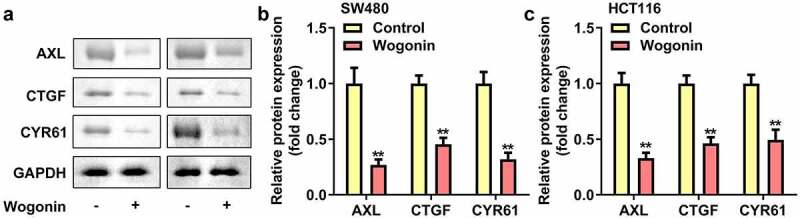
Effect of wogonin on the protein expression of AXL, CYR61 and CTGF in CC cells.

AXL, CYR61 and CTGF was the downstream targeted genes of YAP1. Therefore we explored the protein expressions of AXL, CYR61 and CTGF in CC cells after wogonin treatment. We found that the protein expressions of AXL, CYR61 and CTGF were significantly down-regulated after wogonin treatment (Figure 4).

(a–b) The protein expressions of AXL, CYR61 and CTGF in CC cells were detected by Western blot after wogonin treatment. **P < 0.01, vs. Control group.

### Knockdown of IRF3 decreased YAP1 protein expression in CC cells

**Figure 5.**
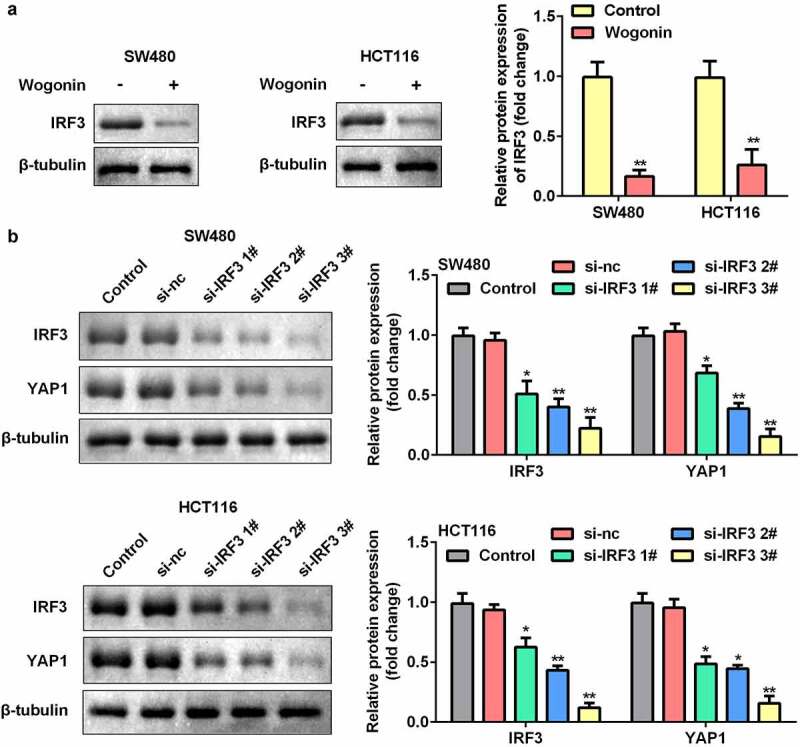
Effect of IRF3 knockdown on the expression of YAP1 in CC cells.

We further explored the function of IRF3 in CC cells. The results showed that the protein expression of IRF3 significantly decreased after wogonin treatment (Figure 5a). In addition, we found that the protein expression of IRF3 was significantly downregulated after si-IRF3 transfection, and knockdown of IRF3 significantly decreased the protein expression of YAP1 (Figure 5b).

(a) The protein expression of IRF3 was measured by Western blotting after wogonin treatment. (b) After si-IRF3 transfection, the protein expression of IRF3 and YAP1 was determined by Western blotting. *P < 0.05, **P < 0.01, vs. Control group.

### Wogonin suppressed IRF3 expression in the nucleus of CC cells

**Figure 6.**
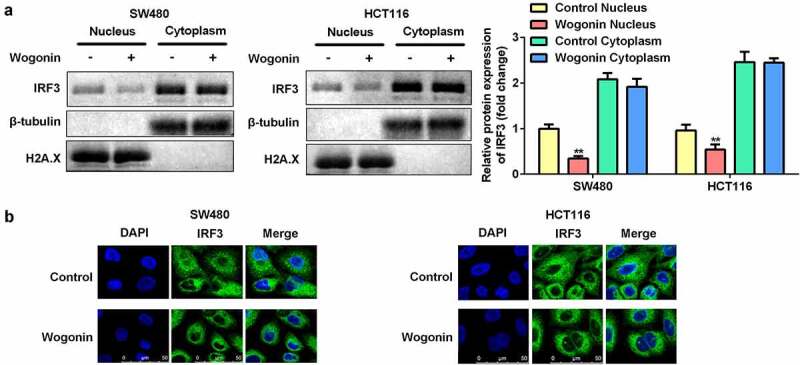
Wogonin suppressed IRF3 translocation to the cell nucleus.

To further investigate the mechanism by which wogonin restricted the function of IRF3, we performed nuclear/cytoplasmic fractionation assays and immunofluorescence staining. After wogonin treatment, the protein expression of IRF3 was significantly decreased in the nucleus, while there was no significant difference in its expression in the cytoplasm (Figure 6a). Immunofluorescence staining showed results similar to that of the fractionation assay (Figure 6b).

(a) The protein expression of IRF3 was measured in the nuclear and cytoplasmic fractions of CC cells treated. (b) Immunofluorescence staining was performed to observe the localization of IRF3. **P < 0.01, vs. Control Nucleus group.

### Overexpression of YAP1 reversed the effects of wogonin

**Figure 7.**
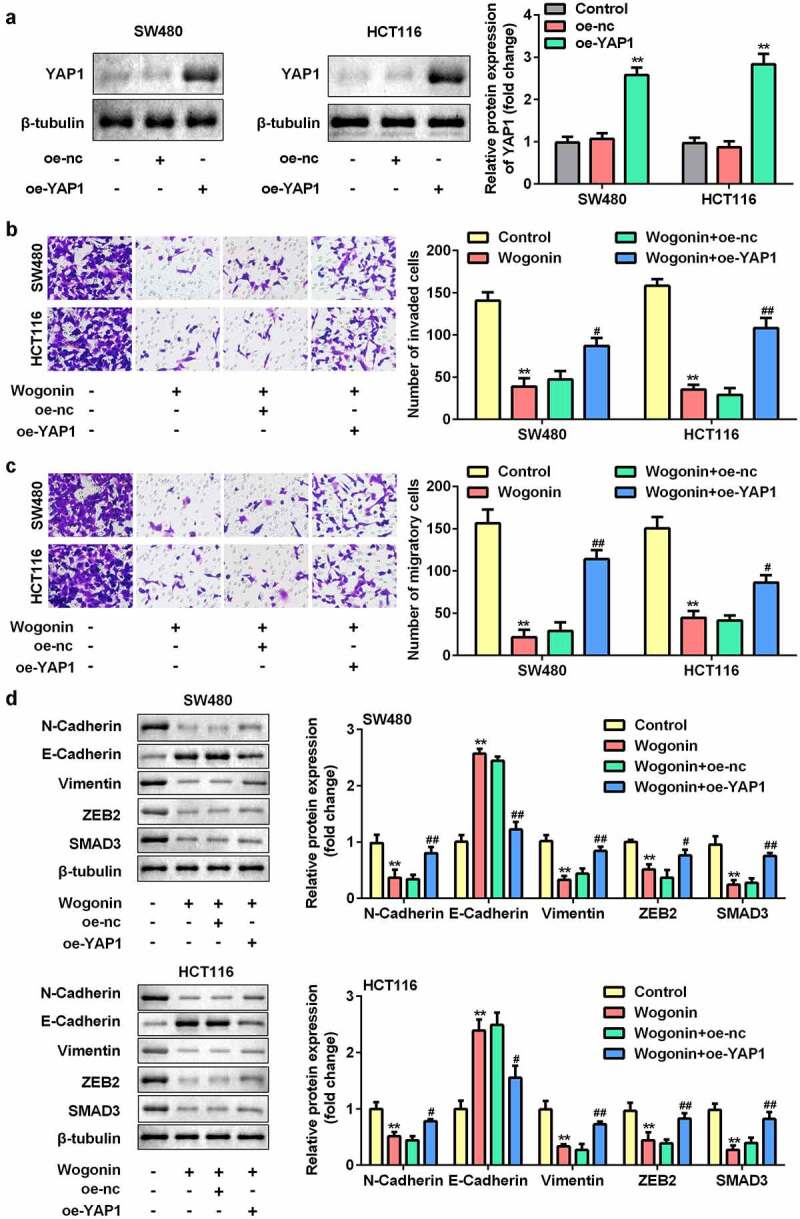
Effects of YAP1 overexpression on the metastasis and protein expression of EMT-related genes in CC cells.

Subsequently, we explored the effect of YAP1 overexpression on metastasis and protein expression of EMT-related genes in CC cells. After oe-YAP1 transfection, the protein expression of YAP1 was significantly increased (Figure 7a). Additionally, we found that overexpression of YAP1 reversed the effects of wogonin on migration, invasion (Figure 7b–c), and the protein expression of E-cadherin, vimentin, ZEB2, N-cadherin, and SMAD3 (Figure 7d).

(a) After oe-YAP1 transfection, the protein expression of YAP1 was tested by Western blotting. (b–c) After oe-YAP1 and wogonin treatment, the invasion and migration abilities of CC cells were measured using the Transwell assay. (d) After oe-YAP1 and wogonin treatment, the protein expression of E-cadherin, vimentin, ZEB2, N-cadherin, and SMAD3 were determined by Western blotting. **P < 0.01, vs. Control group. #P < 0.05, ##P < 0.01, vs. Wogonin+oe-nc group.

### Overexpression of YAP1 reversed the effect of wogonin on weight, volume, and YAP1 expression of the xenograft tumors

**Figure 8.**
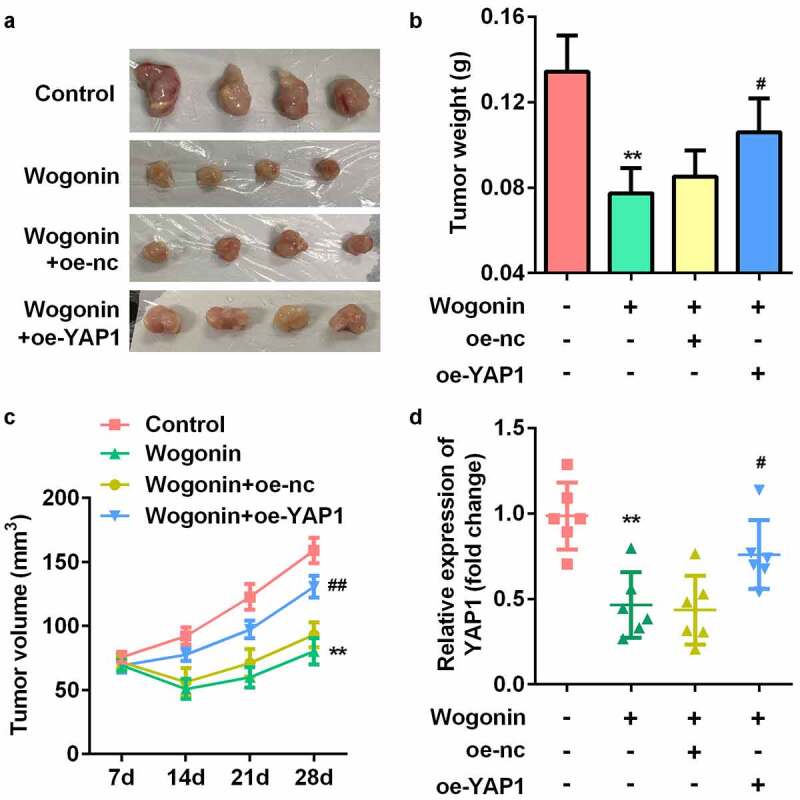
YAP1 reversed the effect of wogonin on weight, volume, and YAP1 expression of the xenograft tumors.

Finally, to explore whether wogonin decreased YAP1 expression *in vivo*, we constructed a subcutaneous transplantation tumor model in BALB/c nude mice (Figure 8a). The results showed that the weight and volume of the tumor were significantly decreased after wogonin treatment, while overexpression of YAP1 reversed this effect (Figure 8b–c). We also found that the mRNA expression of YAP1 was significantly downregulated after wogonin treatment, while overexpression of YAP1 reversed this effect (Figure 8d).

(a) Xenograft tumors of BALB/c female nude mice. (b) The weight of xenografted tumors was measured at 28 days. (c) The volume of the xenografted tumors was measured at 7, 14, 21, and 28 days. (d) The mRNA expression of YAP1 in xenografted tumors was detected by RT-qPCR. **P < 0.01, vs. Control group. #P < 0.05, ##P < 0.01, vs. Wogonin+oe-nc group.

## Discussion

In the current study, we demonstrated that wogonin inhibits EMT development and relieves the carcinogenic process of CC through the IRF3-mediated Hippo signaling pathway *in vivo* and *in vitro*. Our results showed, for the first time, that wogonin possesses anti-tumor effects and exhibits a new regulatory mechanism of YAP1 expression in CC cells.

In recent years, natural compounds extracted from fruits, vegetables, and medicinal plants have attracted extensive attention owing to their anticancer effects and low toxicity. Wogonin is a flavonoid isolated from the roots of the medicinal herb SBG [30]. Previous studies have shown that wogonin specifically induces tumor cell apoptosis and inhibits angiogenesis [31,32]. In ovarian cancer, wogonin has been confirmed to inhibit proliferation and promote apoptosis in dose- and time-dependent manners [13]. Similarly, in lung cancer, wogonin significantly increased the apoptosis rate of A427 cells [33]. However, to the best of our knowledge, there are no reports on the effects of wogonin in CC. In this study, we found that wogonin inhibited the growth and metastasis of CC cells, indicating that wogonin is a potential drug for CC treatment.

Studies have found that the anti-tumor effect of wogonin is closely related to a variety of signaling pathways [30]. The Hippo signaling pathway was first discovered in flies and was also found in homologous molecules of mammals. YAP is the main effector molecule of the Hippo pathway [18]. This pathway controls organ size by regulating cell proliferation and apoptosis. Abnormal regulation of the Hippo signaling pathway causes uncontrolled proliferation and inhibits apoptosis, eventually leading to a series of diseases, including malignant tumors [34]. Many studies have confirmed that YAP may be a candidate oncogene. At the cellular level, YAP overexpression was associated with the loss of cell contact inhibition [34], EMT development [35], anchor-independent growth [36], and other carcinogenic characteristics. At the same time, the carcinogenic effect of YAP has been verified in a variety of animal models [37,38]. In line with these results, we found that wogonin significantly reduced YAP1 expression and improved the phosphorylation level of YAP1. Meanwhile, wogonin enhanced epithelial characteristics and suppressed the acquisition of mesenchymal features, which was similar to the results reported by Lamar et al. [39]. In addition, overexpression of YAP1 reversed the effects of wogonin. Our results indicated that YAP, as a candidate carcinogen, plays an important role in tumorigenesis and progression of CC, and wogonin suppresses EMT development and relieves the carcinogenic process of CC by inhibiting the Hippo signaling pathway.

IRF3 is a well-known signaling mediator and transcription factor. Recent studies have shown that IRF3 is also involved in a wide range of pathophysiological diseases, such as cancer [40], myocardial fibrosis [41], metabolic disorders [42], and so on. A previous study revealed that there is a positive correlation between the expression of IRF3 and YAP, indicating a vital physiological role of IRF3 in YAP activation. Meanwhile, IRF3 overexpression promoted YAP-dependent tumor development [22]. Similarly, we confirmed that IRF3 expression was downregulated after wogonin treatment and knockdown of IRF3 downregulated YAP1 expression. Our results also demonstrated that wogonin may inhibit YAP1 expression by regulating IRF3 expression. In addition, we confirmed that wogonin deactivates IRF3 in the nucleus. Studies have illustrated that once YAP enters the nucleus, it combines with the downstream transcription factor TEAD4, forming a transactivation complex to control target gene expression [18]. Excessive IRF3 is beneficial for the combination of YAP and TEAD4, thus promoting cell growth and development [22]. Overall, our results demonstrated that wogonin inhibits the Hippo signaling pathway by suppressing IRF3 expression in the nucleus.

## Conclusion

In summary, this study confirmed that wogonin inhibited YAP1 expression *in vivo* and *in vitro*. Wogonin suppresses EMT development and the carcinogenic process of CC through the IRF3-mediated Hippo signaling pathway. Our results provide novel insights into the promotion and application of wogonin, which is expected to be a novel therapy for the treatment of CC.

## Article highlights

1. Wogonin inhibited the survival and metastasis of CC cells.
2. Wogonin regulated the IRF3-mediated Hippo signaling pathway.
3. YAP1 overexpression reversed the wogonin effects.
